# Survival Analysis of *Bactrocera oleae* Starvation Resistance During Senescence: The Interactive Influence of Diet, Mating Status, and Sex

**DOI:** 10.3390/insects17030296

**Published:** 2026-03-09

**Authors:** Evangelia I. Balampekou, Thomas M. Koutsos, Dimitrios S. Koveos, Nikos A. Kouloussis

**Affiliations:** 1 Laboratory of Applied Zoology and Parasitology, School of Agriculture, Aristotle University of Thessaloniki, 54124 Thessaloniki, Greece; evibal@agro.auth.gr (E.I.B.); koveos@agro.auth.gr (D.S.K.); 2 Laboratory of Remote Sensing, Spectroscopy, and Geographic Information Systems (GIS), School of Agriculture, Aristotle University of Thessaloniki, 54124 Thessaloniki, Greece; tkoutsos@agro.auth.gr

**Keywords:** aging, longevity, pest control, olive groves, insects physiology

## Abstract

Starvation resistance is a critical fitness trait for the olive fruit fly, *Bactrocera oleae* (Rossi) (Diptera: Tephritidae), influencing survival and reproductive success in fluctuating environments. This study investigates how age (15, 30, and 45 days), diet (full vs. restricted), and mating status (virgin vs. mated) affect the species’ capacity to withstand food deprivation. A multifactorial experiment was conducted on 3600 adults, with survival data analyzed using Kaplan–Meier curves, Log-Rank tests, and Tukey’s HSD. The findings indicate that 15-day-old individuals generally exhibit superior starvation resistance compared to older cohorts. However, these patterns vary by sex and nutritional history. In virgin males on a restricted diet, resistance does not decline linearly with age, while in mated males on a full diet, age has no significant influence on survival. Conversely, in mated females on a full diet, a significant decline in resistance is observed only between the ages of 15 and 45 days. Additionally, for mated adults on a restricted diet, age was found to have a significant influence on survival. These results suggest that starvation resistance in *B. oleae* is not merely a function of chronological age but emerges from complex resource allocation trade-offs between somatic maintenance and reproductive investment.

## Simple Summary

The survival of the olive fruit fly in environments where food is scarce is a critical factor for the spread and reproduction of this major olive cultivation pest. This study investigated how age, diet quality, and mating status affect the insect’s ability to withstand starvation. By examining thousands of adult insects, we found that young individuals aged 15 days are generally more resistant to food deprivation compared to older ones. However, this resistance does not depend on age alone; it is significantly influenced by sex, the type of food consumed, and whether the insect has mated. For example, in mated females, resistance decreases significantly as they grow older, whereas in certain categories of males, age plays a smaller role. The results show that the olive fruit fly manages its energy reserves by making trade-offs between its own survival and its reproductive efforts. Understanding these mechanisms is particularly valuable for researchers, modelers, and practitioners specialized in olive fruit fly management and population dynamics, as it helps in predicting insect populations and designing more effective and targeted methods for protecting olive production.

## 1. Introduction

The olive fruit fly, *Bactrocera oleae* (Rossi) (Diptera: Tephritidae), is widely recognized as the most devastating pest of olive cultivation globally [1]. Its economic impact is primarily driven by larval feeding on the olive mesocarp, which directly reduces crop yield and compromises oil quality. *Bactrocera oleae* is a highly specialized monophagous species whose life cycle is intrinsically linked to the phenology of the olive fruit (*Olea europaea*) [2]. Beyond its narrow host range, this species exhibits significant physiological plasticity; for instance, in the absence of suitable oviposition substrates or under nutritional deprivation, females can reabsorb developed oocytes. This process of oosorption allows for the reallocation of critical limiting resources toward somatic maintenance, potentially extending survival during periods of environmental stress [3,4]. Given the increasing limitations on chemical insecticide use and the demand for sustainable agriculture, Integrated Pest Management (IPM) strategies—including biological control and the Sterile Insect Technique (SIT)—have gained prominence [2]. The success of these strategies, however, relies heavily on a profound understanding of the fly’s physiological resilience and life–history trajectories under fluctuating environmental conditions [2,5,6].

A critical determinant of fitness in tephritid fruit flies is starvation resistance [7]. In the field, adults often encounter periods of nutritional scarcity, where survival depends on the mobilization of metabolic reserves accumulated during the larval stage or early adult life [2,6]. According to the “Teneral Reserve” hypothesis, young adults possess a safety “cushion” of triglycerides in their fat bodies, which provides early vigor but is gradually depleted as the insect ages; in the genus *Bactrocera*, lipid dynamics follow patterns similar to those of other members of the Tephritidae family, where reserves peak early in adult life, serving as a critical buffer against environmental stressors [8]. As adults mature, they must balance the allocation of these limited resources between somatic maintenance and reproductive investment.

This resource allocation is best explained through the lens of the “Disposable Soma” theory [9]. This evolutionary framework posits that organisms’ partition energy between self-repair and reproduction; a high investment in current reproduction often comes at the expense of future survival and stress tolerance [10]. In *B. oleae*, adult nutrition—specifically the availability of protein—is a double-edged sword; while protein intake is essential for maximizing fecundity and oogenesis, it may also accelerate physiological senescence and diminish the fly’s ability to withstand sudden nutritional stress [11,12,13].

The present study builds upon our group’s previous research [7], which provided an initial assessment of starvation resistance in *B. oleae* using standard analysis of variance (ANOVA) across four age groups. In this manuscript, we significantly extend that work by adopting a robust Survival Analysis framework focused on three distinct age cohorts (15, 30, and 45 days). By utilizing Kaplan–Meier curves and Log-Rank tests, we move beyond the comparison of mean survival times to analyze time-to-death distributions and survival probabilities. This methodology allows for a high-resolution mapping of the fly’s physiological resilience, uncovering complex interactions between age, nutritional history, and mating status that remain hidden under traditional statistical approaches. To date, the interaction between chronological age and starvation resistance, particularly across varying dietary and mating histories, represents a complex and unresolved dimension in the physiological ecology of this species.

Starvation resistance has been extensively studied in several insects, most notably in *Drosophila*, providing a robust framework for understanding survival strategies under nutritional stress. Survival in such environments is often dictated by nutrient-sensing mechanisms that regulate longevity [14] and the strategic mobilization of energy reserves [15]. A central component of this response is the evolutionary trade-off between reproduction and survival, where resource allocation toward reproductive effort can significantly diminish resistance to environmental stressors [16,17]. Despite the plasticity of these traits, the potential for further evolutionary adaptation to extreme shifts may be limited by underlying genetic constraints [18], highlighting the need to investigate these responses in economically important species such as *B. oleae*.

The primary objective of this study was to explore the relative impacts of age, diet, and mating status on the starvation resistance of *B. oleae*. Using a multivariable Cox Proportional Hazards model, we estimated how mortality risk fluctuates across three age cohorts (15, 30, and 45 days) for each treatment combination. This approach provides a predictive understanding of survival capacity relative to senescence by quantifying the percentage change in hazard ratios between age groups. By employing an individual-rearing protocol to eliminate social interference [7], we provide a high-resolution mapping of the physiological costs associated with aging and reproductive investment.

## 2. Materials and Methods

### 2.1. Insect Rearing and Maintenance

The study was conducted (from October 2024 till March 2025) at the Laboratory of Applied Zoology and Parasitology in Agriculture, School of Agriculture in Aristotle University of Thessaloniki, Greece. *B. oleae* adults were successfully reared and maintained under stable laboratory conditions in custom-made insect cages based on the model of BugDorm cages (Model DP1000B) usually used in entomological experiments [19]. The conditions were set at a temperature of 25 ± 2 °C, a relative humidity of 45 ± 5%, and a photoperiod of L14:D10 (14 h light:10 h dark). Larvae were reared in (non-infested) olive fruit (‘Chondrolia Chalkidikis’ variety) that had been collected from the wild from olive trees certified to be free of pests and diseases. To minimize inter-individual variability, fruits were standardized by selecting only those within a specific size range (large-fruited variety; average weight 6–10 g, length 25–30 mm). The experiment began with approximately 20,000 adult flies. Following natural mortality, the final number of insects participating in the study was 3600 flies (males and females). In all cases, adults were carefully transferred using a mouth aspirator as a non-invasive means of transportation. Attention was paid to ensure the aspirator acted as a simple transfer tube, allowing insects to enter and exit by walking rather than being forcibly sucked. Any insect that showed any signs of disturbance or injury during the transfer process was immediately removed from the treatment and replaced to ensure enough insects for the experiment.

The rearing process began by collecting infested olive fruits from trees and placing them inside custom-made, wood-framed insect cages. Upon emergence, adult flies were immediately provisioned with a protein-rich diet (enzymatic yeast hydrolysate in a 5:4:1 ratio of water, sugar, and yeast) and provided with continuous hydration via a wetted cotton wick. Tap water was used throughout all experimental phases, including the preparation of diets and hydration during starvation assays. To ensure the absence of confounding factors, the water quality was strictly monitored; it was verified as potable and of high quality, conforming to the safety standards for human consumption, thus ensuring no adverse effects on the physiological performance of the insects.

Once the initial flies emerged, the source olives were removed and replaced with fresh, non-infested olives (previously wild-collected and refrigerated) for mating and oviposition. To prevent larval over-infestation and ensure standardized teneral reserves in the offspring, a controlled infestation protocol was followed. Specifically, larval density was restricted to a maximum of two oviposition stings (oviposition punctures) per olive fruit. This was achieved by leaving the olives inside the cages for two hours approximately. Adult cohorts were maintained in standard cages (size: 20 × 20 × 20 cm^3^) at a density of 10 individuals, with a 1:1 male-to-female ratio to standardize social and reproductive interactions prior to the commencement of the starvation assays. The subsequent egg-infested olives were quickly transferred from the cages into covered basins, where suitable cloth maintained optimal humidity and temperature for larval development. After the larvae pupated, the pupae were moved into 20 × 20 × 20 cm^3^ plexiglass cages just prior to their final emergence, and the resulting adults were then maintained on one of two controlled regimes: a full diet (protein, sugar and water) or a restricted diet (sugar and water).

### 2.2. Experimental Design

**Figure 1:**
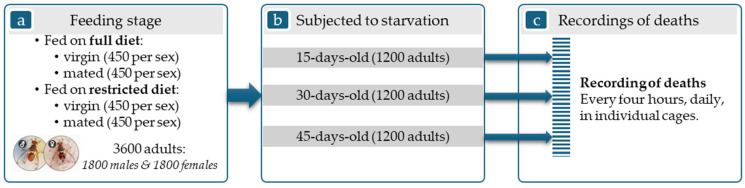
Experimental process: (**a**) Feeding stage; (**b**) Starvation stage; (**c**) Recording deaths.

An experiment on the starvation resistance of the olive fruit fly was conducted under controlled laboratory conditions and assessed the combined effects of the factors: Diet (2 levels: Full [5:4:1 water/sugar/yeast ratio] and Restricted [sugar-only]), Mating Status (2 levels: Virgin and Mated), Sex (2 levels: Male and Female), and Age (3 cohorts: 15, 30, and 45 days). A total of 3600 insects (150 individuals per cohort placed in 15 cages with 10 individuals in each cage, therefore a total number of 360 plexiglass cages were used) were subjected to a multi-factor experiment involving eight treatments in three age groups. In each plexiglass having insects for mating, 5 males and 5 females were placed together. The goal is to have always a maximum number of 10 insects per cage. Figure 1 depicts the three main stages of the experimental process: (a) the feeding stage (treatments with insects fed on a full or restricted diet); (b) adults of 3 age groups subjected to starvation; (c) recording deaths every 4 h, daily, in their individual cages. 

### 2.3. Starvation Experimental Protocol

#### 2.3.1. Procedure for Setting Experimental Insects to Starvation

The experimental protocol was initiated by placing pupae in Petri dishes within large plexiglass enclosure units (20 × 20 × 20 cm^3^). Upon emergence, adults were isolated and housed individually in truncated cone-shaped plastic cages with a base diameter of 9.5 cm, a top diameter of 6.5 cm, and a total height of 12.5 cm (effective internal height of 8 cm). Within these individual units, subjects were provisioned with water and assigned to either a full or restricted dietary regime, ensuring precise monitoring of nutritional intake and survival at the individual level. The experimental design consisted of 150 independent replication units per treatment combination (24 treatments × 150 individual cages = 3600 individual cages). Thirteen days of post-emergence, to attain successful mating, the flies were temporarily transferred to larger 20 × 20 × 20 cm^3^ cages for a 48-h period (fed on the corresponding diet). Two distinct setups were adopted: single-sex groups (ten virgin males or ten virgin females per cage) and mixed-sex groups (five virgin males and five virgin females per cage) to ensure mating, while strictly maintaining the assigned dietary regimes. Following this 48-h window, the flies were promptly returned to their individual cages to eliminate confounding effects from crowding or social interactions. Mating success was verified via direct observation between 16:00 and 21:00, coinciding with the species’ peak natural courtship period [20]. Any individuals that failed to mate were excluded and replaced with verified, mated flies to ensure experimental consistency.

#### 2.3.2. Observation During the Starvation Period and the Recording of Deaths

Upon emergence, each insect was housed in an individual plastic cage (3600 units total). For a 48-h window, insects were grouped in the 20 × 20 × 20 cm^3^ plexiglass cages (15 replicates × 10 individuals × 24 experimental combinations), totaling 3600 individuals. After this window, the insects were then housed in individual plastic cages with the corresponding diet. By the time the insects reached the age to be subjected to starvation (15, 30 and 45 days), each insect was finally transferred to a new clear individual plastic cage provided only with water. Mortality was monitored daily at 4-h intervals throughout the light phase of the photoperiod (08:00, 12:00, 16:00, and 20:00) until all individuals had expired. To confirm death, a fine paintbrush was used to gently prod any inactive insects. Furthermore, all plastic cages were rotated daily to minimize potential positional biases within the laboratory environment. Finally, starvation resistance was quantified as the time elapsed from the initiation of starvation to the confirmed time of death for everyone.

### 2.4. Statistical Analysis

Starvation resistance was first analyzed separately for each sex using a three-way Analysis of Variance (ANOVA) within the General Linear Model (GLM) framework. Mating status (virgin, mated), diet (full, restricted), and age (15, 30, 45 days) were treated as fixed factors, including all two-way and three-way interactions. Post hoc comparisons were conducted using Tukey’s HSD test [21]. Model assumptions were verified through visual inspection of residual plots and formal testing, including Kolmogorov–Smirnov for normality and Spearman’s rho for homoscedasticity. Given the exceptionally large sample size (*N* = 1800 adults per sex, therefore 3600 insects in total), the ANOVA framework was deemed robust to potential violations of the normality assumption, as the sampling distribution of the mean approximates normality regardless of the underlying data distribution according to the Central Limit Theorem [22,23]. This ensures the reliability of the F-statistics and provides maximum statistical power (1.000) [24].

To further account for the dynamic nature of mortality, a Survival Analysis framework was employed. Survival probabilities were estimated via Kaplan–Meier curves [25], with differences between cohorts evaluated through the Log-Rank (Mantel–Cox) test [26]. To account for the inflation of Type I error due to multiple comparisons across the eight treatment groups, a Bonferroni correction was applied. The significance threshold was adjusted to *α* = 0.00625 (0.05/8 comparisons).

Additionally, a Cox Proportional Hazards Model was used to quantify the relative contribution of each factor to the risk of death by estimating Hazard Ratios (HR) [27]. For the Cox proportional hazards regression analysis, the ‘Enter’ method was utilized for variable inclusion. Age was treated as the primary independent predictor within each of the eight biological strata (Sex × Diet × Mating Status). To obtain a comprehensive evaluation of the aging effect, pairwise comparisons were performed among all age cohorts (15, 30, and 45 days). Specifically, the 15-day group served as the initial reference category to calculate Hazard Ratios (HR) for the 30 and 45-day cohorts, while additional models were executed using the 45-day group as the reference to allow for direct comparison with the 30-day cohort. The overall significance of the model coefficients was verified through the Omnibus Test. The proportional hazards assumption was verified using Schoenfeld residuals [28]. Statistical significance for all hypothesis tests was evaluated at a threshold of *α* = 0.05 (*p* ≤ 0.05). All analyses were performed using IBM SPSS Statistics v.29.0.

## 3. Results

### 3.1. Descriptive Statistics

Table 1 summarizes the mean lifespan (days) of *B. oleae* across all experimental treatments. A consistent physiological decline in survival probability is identified as a function of chronological age across all experimental groups. Individuals within the 15-day-old cohort exhibited the highest survival rates, significantly outperforming the 30-day and 45-day age groups regardless of sex, dietary regimen, or mating status. For instance, among virgin females on a full diet, the mean survival at 15 days was 72.2 ± 2.1, compared to 56.0 ± 2.5 and 49.5 ± 1.5 at 30 and 45 days, respectively. Similarly, full-diet virgin males showed a marked reduction from a mean of 72.2 ± 3.8 at 15 days to 50.4 ± 3.0 at 30 days and 43.6 ± 2.3 at 45 days. This downward trend is further substantiated by the non-overlapping 95% confidence intervals between the 15-day cohort and subsequent age stages in most treatments, suggesting that the initial post-maturation period represents a window of peak vigor before senescence or cumulative physiological stressors significantly diminish survivorship.

### 3.2. Statistical Analysis: Log-Rank (Mantel–Cox) and Kaplan–Meier Survival Curves

To determine the statistical significance of age-related differences in survival, Log-Rank (Mantel–Cox) tests were performed across all eight experimental treatments. The results indicate a general effect of age (Table 2): in six out of the eight factor combinations, age had a highly significant impact on starvation resistance. This suggests that resistance decreases significantly as the insect ages. Two exceptions were observed in males: (a) the effect of age was not statistically significant in mated males on a full diet, and (b) it was similarly non-significant in virgin males on a restricted diet. These exceptions suggest that, under specific environmental and physiological conditions in males, the ‘cost’ of aging may be mitigated and thus masked by other factors, such as the high energetic demands of mating or an existing baseline of low starvation resistance. In females, however, the effect of age remained highly significant regardless of diet or mating status, confirming a robust correlation between senescence and reduced starvation resistance.

Statistically significant differences in survival were observed across most treatments (Table 2). Notably, these differences remained highly significant even after applying the Bonferroni correction for multiple testing (*p* < 0.00625 in most cases), indicating that the observed effects are robust and not due to chance.

**Figure 2:**
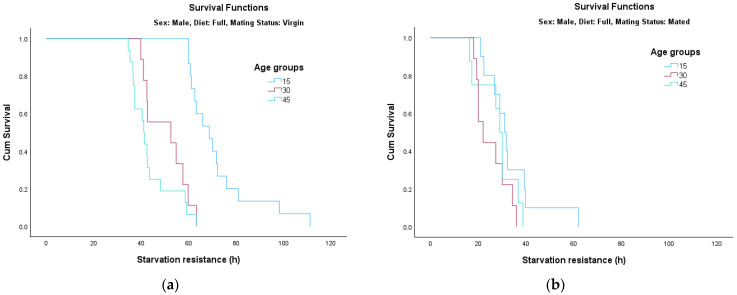
Survival curves for male adults fed on full diet: (**a**) virgin; (**b**) mated.

**Figure 3:**
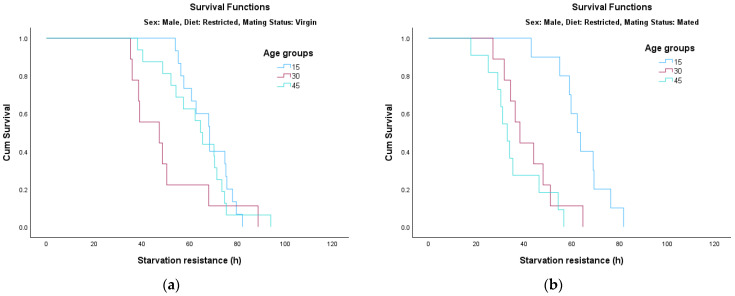
Survival curves for male adults fed on restricted diet: (**a**) virgin; (**b**) mated.

**Figure 4:**
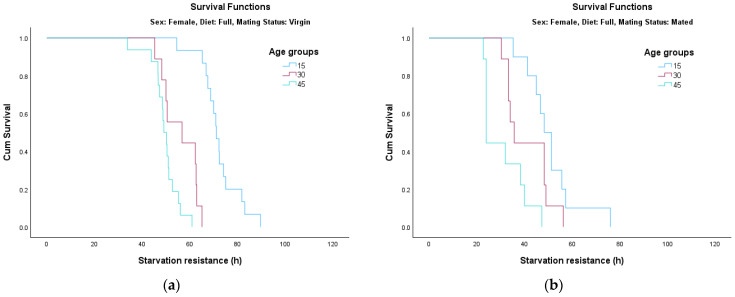
Survival curves for female adults fed on full diet: (**a**) virgin; (**b**) mated.

**Figure 5:**
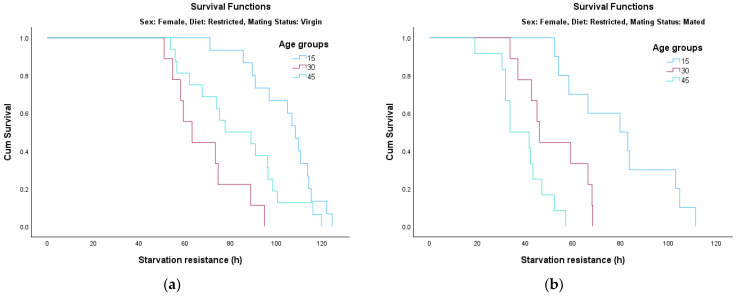
Survival curves for female adults fed on restricted diet: (**a**) virgin; (**b**) mated.

The analysis of survival functions (Survival Curves, Figure 2, Figure 3, Figure 4 and Figure 5) reveals distinct patterns of starvation resistance, which are modulated by age cohort (15, 30, and 45 days) in relation to diet and mating status in both sexes. A consistent trend observed across most experimental conditions is a negative correlation between age and starvation resistance. The age groups are represented as follows: the 15-day-old cohort is indicated by the blue line, the 30-day-old cohort by the pink/red line, and the 45-day-old cohort by the green line.

The following survival dynamics were identified for males and females:

*Survival Dynamics in Males:*

- In male cohorts, starvation resistance generally exhibited a negative correlation with chronological age.
- Full Diet (Figure 2a): For virgin males fed on a full diet, age had a highly significant impact on survival (*χ*^2^ = 32.608, *df* = 2, *p* < 0.001).
- Full Diet (Figure 2b): In contrast, for mated males on the same diet, the differences between age groups were not statistically significant (*p* = 0.123); mating appeared to reduce survival across all ages to a similarly low baseline.
- Restricted Diet (Figure 3a): Under a sugar-based diet, virgin males showed no significant age-dependent variation in survival (*p* = 0.149).
- Restricted Diet (Figure 3b): However, once mated, age became a highly significant factor (*χ*^2^ = 18.471, *df* = 2, *p* < 0.001), with younger cohorts (15 days) showing markedly better resistance than older groups.

*Survival Dynamics in Females*

Female cohorts followed a consistent and highly significant age-dependent decline in starvation resistance across all experimental treatments (*p* < 0.001 in all cases).

- Full Diet (Figure 4a): Age group differences were most pronounced in virgin females (*χ*^2^ = 39.808, *df* = 2, *p* < 0.001), with a sharp decrease in resistance as age increased from 15 to 45 days.
- Full Diet (Figure 4b): The age-related decline remained highly significant in mated females as well (*χ*^2^ = 15.256, *df* = 2, *p* < 0.001).
- Restricted Diet (Figure 5a): Dietary restriction appeared to exert a protective effect, particularly for younger females; virgin females at 15 days exceeded 120 h of survival.
- Restricted Diet (Figure 5a,b): Statistical analysis confirmed that age significantly influenced survival for both virgin (*χ*^2^ = 19.439, *df* = 2, *p* < 0.001) and mated (*χ*^2^ = 23.858, *df* = 2, *p* < 0.001) females.

The analysis reveals a consistent “mating cost” across both sexes. Virgin individuals generally outperformed mated individuals in starvation endurance, regardless of diet. This disparity suggests a critical trade-off, where reproductive investment is likely to deplete energy reserves otherwise allocated to somatic maintenance. While dietary restriction helped mitigate some of this cost—especially in females—aging remained the dominant factor in determining survival probability under starvation conditions.

### 3.3. Multivariable Risk Assessment (Cox Proportional Hazards Model)

To quantify the independent effects of age, sex, diet, and mating status on the hazard of mortality during food deprivation, a multivariable Cox Proportional Hazards Model was employed. The proportionality assumption was verified using Schoenfeld residuals and found to be valid for all covariates. The Cox analysis confirmed that age is a substantial factor determining resistance to food deprivation across both sexes and all dietary regimens. The analysis focused on statistically significant pairwise comparisons (*p* ≤ 0.05).

Depending on which age group is used as reference, we can elucidate critical information based on the Cox proportional hazards analysis as follows:

- *Age-Dependent Survival Patterns (Reference Age: 15 Days):*

When 15-day-old individuals were used as the reference group, the Cox proportional hazards analysis revealed a significant age-related increase in mortality risk across most treatment combinations (Table A1—Appendix A). Among virgin males on a full diet, mortality risk rose sharply with age; 30- and 45-day-old individuals exhibited 7.4- and 12.9-fold higher hazards of death, respectively, compared to the reference group (*χ*^2^ = 24.690, *p* < 0.001). In virgin females on a full diet, the effect of age was even more pronounced, with hazard ratios (*Exp*(*B*)) of 37.9 and 115.3 for 30- and 45-day-olds, respectively (*p* < 0.001), indicating an exponential increase in mortality risk with advancing age. For virgin males on a restricted diet, the increase in mortality was weaker and did not reach statistically significant until 45 days (*Exp*(*B*) = 2.281, *p* = 0.025), suggesting that sugar feeding attenuated the age effect. Conversely, virgin females on a restricted diet displayed a significant but moderate rise in risk with age (*Exp*(*B*) = 7.673 at 30 days; *Exp*(*B*) = 2.352 at 45 days, both *p* < 0.05). Among mated males on a full diet, no significant differences were detected (*p* > 0.1), with hazard ratios remaining below 3, implying that mating may buffer the negative effects of age under protein feeding. In mated females on a full diet, mortality increased significantly with age (*Exp*(*B*) = 7.244 at 30 days and 2.164 at 45 days, *p* = 0.002), reflecting the combined costs of reproduction and protein metabolism. Mated males on restricted diet showed a progressive and significant increase in mortality risk (*Exp*(*B*) = 4.898 at 30 days; *Exp*(*B*) = 9.391 at 45 days, *p* < 0.01), while mated females on the same diet exhibited a steep rise, with hazard ratios of 4.756 and 16.173 at 30 and 45 days respectively (*p* < 0.001). Overall, the 15-day reference model demonstrates that the interaction between age, diet, and sex strongly determines survival outcomes, with the most pronounced mortality increases observed in females maintained on protein diets.

- *Age-Dependent Survival Patterns (Reference Age: 45 Days)*

Using 45-day-old individuals as the reference group produced the inverse trend (Table A1—Appendix A). Younger individuals consistently exhibited significantly lower mortality risks (*Exp*(*B*) < 1), confirming the cumulative increase in death probability with age. In virgin males on a full diet, 15- and 30-day-old individuals showed markedly lower hazards (*Exp*(*B*) = 0.028 and 0.187, respectively; *p* < 0.001), reflecting reduced age-related mortality. Similarly, virgin females on a full diet displayed dramatic reductions in risk (*Exp*(*B*) = 0.009 at 15 days, *p* < 0.001), further confirming the steep age effect identified in the first model. Among virgin males on a restricted diet, younger individuals exhibited reduced risks, although the effect was weaker (*Exp*(*B*) = 0.329 at 15 days, *p* = 0.028). Virgin females on a restricted diet showed a modest but significant reduction in mortality (*Exp*(*B*) = 0.425 at 15 days, *p* = 0.025), suggesting that sugar diets (restricted diet) mitigate age-related decline more effectively than protein diets. In mated males on a full diet, age-related differences were not statistically significant (*p* > 0.2), indicating relative stability in survival probabilities across ages under this condition. Conversely, mated females on a full diet exhibited a substantial decline in mortality risk at younger ages (*Exp*(*B*) = 0.138, *p* = 0.045), confirming that age and reproduction interact to accelerate mortality under protein feeding. For mated males on a restricted diet, hazard ratios decreased significantly at younger ages (*Exp*(*B*) = 0.106 and 0.304 for 15 and 30 days, respectively; *p* < 0.001), while mated females on the same diet again showed a sharp age effect, with *Exp*(*B*) = 0.061 at 15 days (*p* < 0.001), indicating that survival probability drops rapidly with age in this treatment as well.

**Figure 6:**
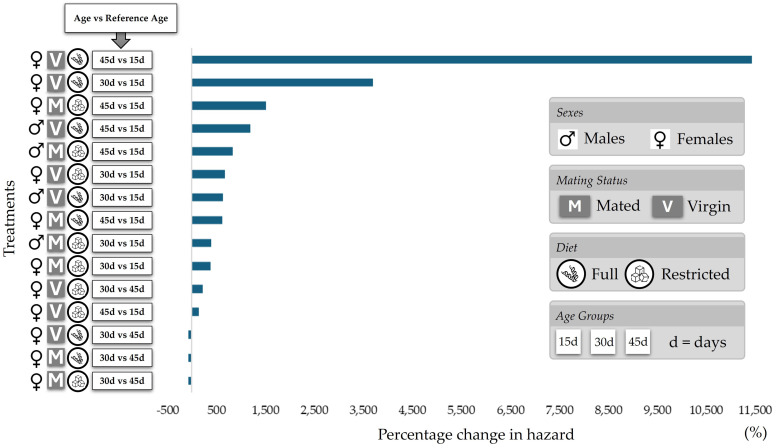
Pairwise comparisons of mortality hazard across age groups. The bar chart illustrates the percentage change in mortality hazard between specific age cohorts (15 d, 30 d, and 45 d) for each treatment combination. Treatments are categorized by sex (males, females), mating status (mated, virgin), and diet (full, restricted). The labels on the *y*-axis indicate the specific age being evaluated against a reference age, which serves as the baseline for each pairwise comparison (e.g., 45 d vs. 15 d). These comparisons are presented alongside the corresponding icons for sex, mating status, and diet.

The impact of aging on mortality dynamics was assessed through pairwise comparisons of mortality hazards across different age cohorts (15 d, 30 d, and 45 d). As illustrated in Figure 6, the percentage change in mortality hazard was calculated for each treatment combination, using a specific age group as the baseline (Reference Age) for each comparison. These comparisons were conducted across all experimental groups, accounting for sex (males and females), mating status (mated and virgin), and dietary conditions (full and restricted diets). To quantify the magnitude of these effects, the column ‘Change (%*Δ*)’ in Table A1 represents the relative percentage change in the hazard of mortality, derived from the estimated regression coefficient (*B*) using the formula below:

(1) $$\%\Delta =(e^{B}-1)\times 100$$

where: $e^{B}$ = *Exp*(*B*), represents the Hazard Ratio (HR).

Aging exerted the most substantial impact on virgin females under a full diet (V_F_♀), where the mortality hazard for 45-day-old cohorts surged by 11,426.3% relative to 15-day-old individuals ($e^{B}$ = *Exp*(*B*) = 115.263, *p* < 0.001). This age-dependent increase was nearly tenfold higher than that recorded for males of the same experimental group (V_F_♂: +1189.6%). Conversely, the markedly smaller percentage shifts observed in males under dietary restriction or post-mating conditions—specifically in 45-day-old virgin males on a restricted diet (V_R_♂), where the mortality risk increased by 23.5% relative to the 15-day reference group, and in 45-day-old mated males on a full diet (M_F_♂), with an 87.0% increase—confirm that nutritional quality and reproductive status can modulate the trajectory of age-related vulnerability. Overall, the pairwise comparisons in Figure 6 and the associated risk indices (Table A1—Appendix A) underscore that while aging universally increases mortality risk, the magnitude of this increase is acutely sensitive to the interaction between sex, mating status, and dietary history.

**Figure 7:**
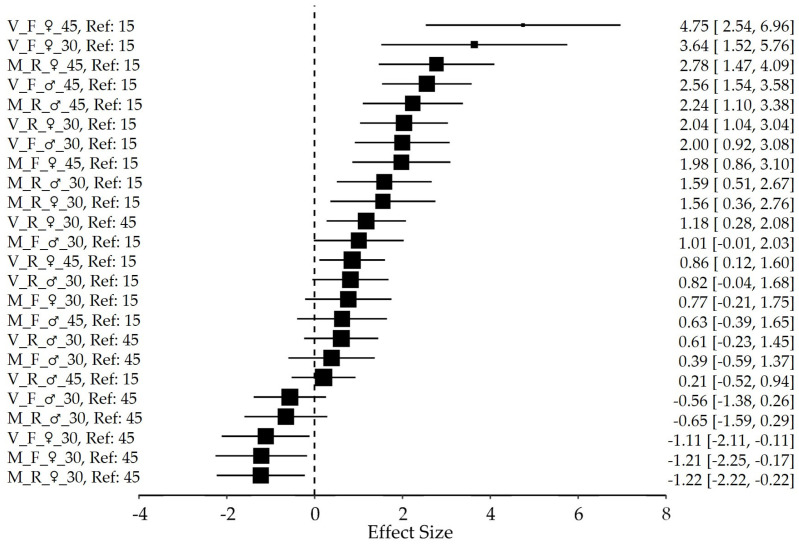
Forest plot of the stratified Cox Proportional Hazards regression analysis. The plot displays the estimated Log-Hazard Ratios (*B*) on the *x*-axis for 24 distinct treatment groups. The Effect Size (*B*) provides a logarithmic measure of the risk, which directly translates to the Change (%*Δ*) in mortality hazard reported in Table A1—Appendix A; for instance, *B* = 4.75 corresponds to a +11,426.3% increase in hazard. Black squares represent the point estimates, where the size of the square is proportional to the statistical weight of the group (inverse of the variance). Horizontal lines indicate the 95% Confidence Intervals (CI). Treatments positioned to the right of the zero-line (*B* > 0) indicate an increased hazard (higher risk), while those to the left (*B* < 0) indicate a protective effect (lower risk). Groups whose 95% CI do not intersect the vertical null line (0) are considered statistically significant at the *p* < 0.05 level. The notation Ref: 15 and Ref: 45 identifies the reference age (15 days or 45 days) against which each treatment group is compared.

To evaluate the relative risk of insect mortality across the treatments, a Cox Proportional Hazards regression was performed on 24 distinct treatment groups. The results, summarized in Figure 7, illustrate the distribution of Log-Hazard Ratios (*B*) and their associated 95% Confidence Intervals (*CI*). Treatments involving virgin individuals on a full diet (V_F) demonstrated the highest positive coefficients. Specifically, 45-day-old virgin females on a full diet compared to the 15-day-old reference group (V_F_♀_45, Ref: 15) exhibited the most pronounced risk (*B* = 4.75, 95% CI [2.54, 6.96]). Conversely, mated individuals on restricted (M_R) or full (M_F) diets generally exhibited negative coefficients, indicating a protective effect. Notably, 30-day-old mated females on a restricted diet (M_R_♀_30, Ref: 45; *B* = −1.22, 95% CI [−2.22, −0.22]) and those on a full diet (M_F♀_30, Ref: 45; *B* = −1.21, 95% CI [−2.25, −0.17])—both compared to their 45-day-old counterparts—showed the most significant hazard reductions relative to the baseline.

Statistical precision, indicated by the weight of the point estimates in the plot, was highest for groups in the center of the distribution, where narrower confidence intervals suggest more stable estimates of the hazard. Groups whose confidence intervals do not intersect the vertical null line (at 0) are considered statistically significant at the *p* < 0.05 level.

While the time-dependent covariate analysis indicated marginal deviations from the proportional hazard’s assumption in two specific strata (*p* = 0.048 and *p* = 0.036, Table A2—Appendix A), the visual synthesis provided by the Forest Plot (Figure 7) confirms that these deviations are minor and do not compromise the biological interpretation. Specifically, the consistent alignment of Hazard Ratios to the right of the null line across all cohorts demonstrates a robust and universal age-related decline in starvation resistance, regardless of the slight temporal variations in hazard rates within those few groups.

Starvation resistance was evaluated relative to age at onset (15, 30, and 45 days) across eight experimental treatments defined by sex, mating status (virgin vs. mated), and dietary history (full vs. restricted diet). The proportional hazards assumption was verified for each stratum by testing the significance of time-dependent covariates (interaction of Age with time). The results of these omnibus tests confirmed the stability of the model across time, and the full diagnostic output is available in (Table A2—Appendix A).

### 3.4. Post Hoc Analysis of Starvation Resistance (Tukey’s HSD)

**Figure 8:**
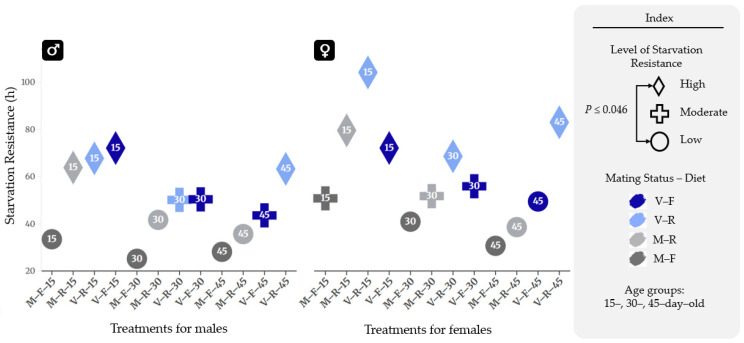
Starvation resistance (in hours) per treatment: Statistically significant differences (*p* ≤ 0.046) were observed between the ‘low’ (circles) and ‘high’ (diamonds) resistance groups. Data points of the same color indicate treatments with identical mating status and dietary regimes. Blue symbols represent virgin flies, whereas gray symbols denote mated individuals. Lighter shades depict the restricted diet regime, while darker shades represent the full diet.

These phenotypic differences in mean survival hours, as identified by ANOVA and Tukey HSD, reinforce the hazard analysis results, providing a multi-layered validation of the physiological impact of age, diet, and mating on *B. oleae* survival. The impact of dietary regimes, mating status, and age on starvation resistance (expressed in hours) is summarized in Figure 8, with the corresponding descriptive statistics presented in Table 1. Statistical analysis revealed distinct groupings based on resistance levels, with high-resistance groups (indicated by diamonds) showing significantly greater survival times compared to low-resistance groups (indicated by circles) (*p* ≤ 0.046). The physiological response was influenced by the interaction of life–history traits: virgin individuals (blue symbols) generally exhibited higher resistance than mated counterparts (gray symbols), while full dietary regimes (darker shades) further modulated these trends across the 15-, 30-, and 45-day-old age groups.

**Figure 9:**
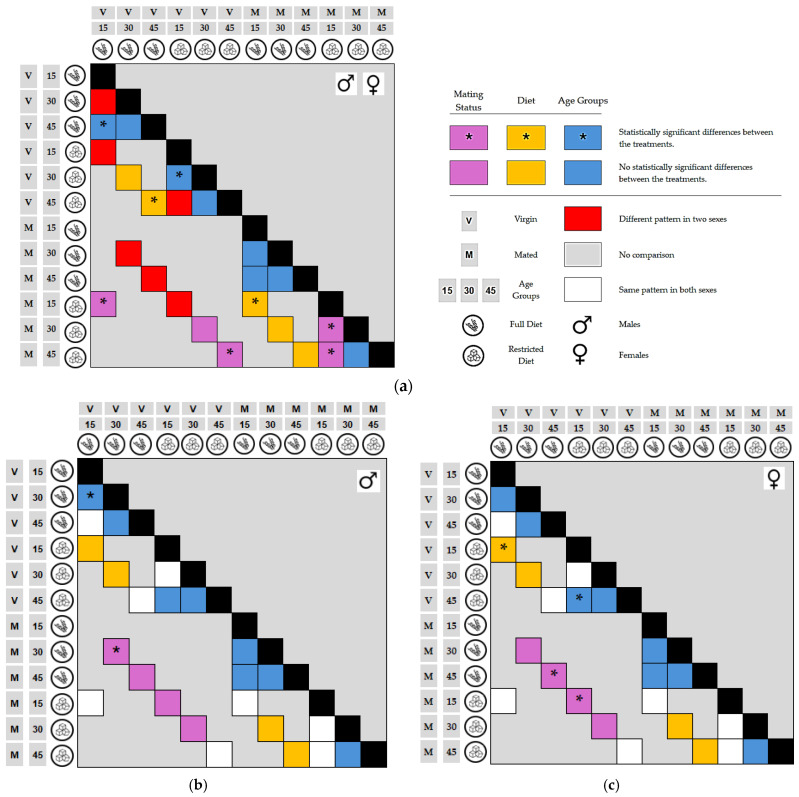
Representation of ANOVA and Tukey HSD results: (**a**) Pairs of comparisons exhibiting statistically significant differences in both sexes simultaneously, with red cells marking divergent overall patterns. (**b**) Comparisons with significant differences observed exclusively in males. (**c**) Comparisons with significant differences observed exclusively in females. Color-coded cells denote the factor compared: yellow for Diet, blue for Age Groups, and purple for Mating Status. White cells in panels (**b**) and (**c**) indicate shared patterns with the opposite sex. Asterisks (*) indicate statistically significant differences between treatments, while solid-colored cells without an asterisk signify no significant difference. Gray cells represent areas where no comparison was performed. Black cells indicate the main diagonal, representing parameter self-correlation.

To further elucidate the statistical significance of these interactions, Figure 8 provides a comprehensive mapping of the ANOVA (Table A3—Appendix A) and Tukey HSD post hoc results through a comparative matrix, based on the detailed data (Table A4—Appendix A). While Figure 8 illustrates the phenotypic values, Figure 9 decodes the specific pairwise comparisons between treatments. Within the matrices, the color-coded cells denote the specific experimental factor under evaluation: yellow represents Diet, blue corresponds to Age Groups (15, 30, and 45 days), and purple denotes Mating Status (virgin vs. mated). Statistically significant differences are explicitly indicated by an asterisk (*), while solid-colored cells without an asterisk represent comparisons that did not reach statistical significance.

The layout of Figure 9 is designed to distinguish between shared and sex-specific responses: panel (a) identifies pairs of treatments where both sexes exhibit identical statistical patterns, whereas panels (b) and (c) delineate sex-specific effects, highlighting the treatment pairs that show significant differences exclusively in males and females, respectively. Additionally, the use of red cells in the matrix for panel (a) marks instances where the overall response pattern differs between the two sexes, while white cells in panels (b) and (c) indicate comparisons that follow a shared pattern in the opposite sex. This visualization offers a detailed overview of the interaction between sex and the studied variables, providing a clear representation of sex-specific physiological plasticity.

Detailed quantitative analysis (Table A4—Appendix A) highlights chronological age as the primary driver of metabolic decline across almost all cohorts. In males, 15-day-old virgin individuals on a full diet survived significantly longer than their 30-day-old (21.749 h, *p* = 0.002) and 45-day-old counterparts (28.554 h, *p* < 0.001). A similar age-dependent reduction was observed in mated males on restricted diet, with 15-day-olds outlasting older groups by more than 22 h. Females exhibited even more acute sensitivity to senescence; for instance, virgin individuals on a restricted diet showed a drastic drop of 35.556 h in resistance between days 15 and 30 (*p* < 0.001). Furthermore, mating status significantly established a lower baseline for resistance early in life, as evidenced by 15-day-old virgin males on a full diet maintaining a 38.660-h advantage over mated individuals (*p* < 0.001).

The robustness of these effects is further supported by the Partial Eta Squared (*η_p_*^2^) values in the Two-Way ANOVA (Table A3—Appendix A), which quantify the effect size of each factor. In females, chronological age and mating status exerted the most substantial influence on survival variance (*η_p_*^2^ = 0.454 and 0.442, respectively), while in males, mating status was the primary determinant of physiological performance (*η_p_*^2^ = 0.421). Furthermore, the Observed Power for all primary factors was 1.000, confirming that the sample size was more than sufficient to detect these high-magnitude biological effects. These high effect sizes confirm that the observed declines in starvation resistance are not merely statistically significant but are the dominant drivers of the fly’s life history.

These findings, which align with the hazard trajectories identified in the Cox models, integrated with the visual transitions from high to low resistance groups in Figure 8 and Figure 9, underscore a fundamental life–history trade-off between reproduction and survival. The results indicate that starvation resistance in *B. oleae* is a depletable trait; in early life, resources are maximized for somatic maintenance, particularly under carbohydrate-rich regimes. However, the physiological cost of reproduction—specifically the diversion of protein toward oogenesis and mating behaviors—accelerates the exhaustion of somatic reserves. This metabolic vulnerability is most pronounced in older, mated cohorts, which consistently cluster into the ‘low resistance’ phenotype. Ultimately, while sugar-loading (restricted diet) can serve as a temporary metabolic buffer, senescence remains the final arbiter of survival, leading to a universal decline in resilience as organisms approach the end of their life cycle.

## 4. Discussion

The survival analysis presented in this study provides a comprehensive characterization of the survival dynamics of *B. oleae* under starvation stress, specifically focusing on the physiological decline associated with senescence. The capacity of tephritid fruit flies to withstand periods of nutrient deprivation is a fundamental determinant of their adaptive success and persistence in heterogeneous landscapes [29]. Our findings demonstrate that starvation resistance in the olive fruit fly is governed by a complex, multi-factorial interplay between chronological age, mating status, and adult dietary quality, aligning with core tenets of insect physiological ecology regarding energy partitioning and life–history trade-offs.

### 4.1. Senescence and Physiological Decay

This investigation establishes age as a primary determinant of survival, with senescence inducing a statistically significant reduction in starvation resistance. The observed decline aligns with the “disposable soma” theory of aging [9,30], which posits that organisms prioritize somatic maintenance during early adulthood to ensure reproductive success, followed by a rapid decline in stress tolerance as cumulative cellular damage and the depletion of endogenous energetic reserves take hold [31].

It was observed that younger adults (15 days old) consistently displayed superior resistance compared to senescent individuals (45 days old). This decline was most dramatic in females on a full diet, where the hazard ratio exhibited a 115-fold increase by day 45. This 115-fold increase, which translates to a +11,426.3% rise in the risk of mortality compared to the 15-day-old cohort (Table A1—Appendix A), underscores the devastating impact of senescence on the physiological integrity of this species. Such age-dependent physiological shifts are inextricably linked to survival probability in tephritids [12,32,33], supporting the hypothesis that the aging process in *B. oleae* compromises the homeostatic mechanisms required to manage acute environmental stressors [34].

The observed survival patterns in *B. oleae* reflect broader physiological and evolutionary principles established in other dipterans. Starvation resistance is a complex trait governed by nutrient-sensing pathways, such as insulin and TOR signaling, which coordinate the allocation of resources between longevity and stress resistance, reflecting the principles of nutritional geometry where the ratio of macronutrients dictates the metabolic shift between survival and fecundity [14]. In this context, the phenotypic decline in resistance we observed with age and reproductive activity likely underscores the high physiological cost of reproduction, a phenomenon where investment in current offspring diminishes the capacity to withstand environmental insults [16]. The biological dominance of these factors is further validated by the high effect sizes observed in our GLM analysis, where age and mating status explained a substantial proportion of the total survival variance (*η_p_*^2^ up to 0.454), confirming that these are the primary drivers of the fly’s life history.

While our study focuses on phenotypic outcomes, the literature suggests that such survival dynamics are fundamentally linked to the efficient mobilization of endogenous reserves and the functional integrity of storage tissues like the fat body [15,17]. However, the ability of these populations to evolutionarily shift their survival thresholds in response to changing environments may be limited; as demonstrated in laboratory evolution experiments, certain physiological limits, particularly those related to thermal and nutritional stress, can be constrained by low evolutionary potential [18]. This highlights the importance of understanding the inherent physiological boundaries of *B. oleae* when predicting its persistence in fluctuating landscapes.

### 4.2. Reproductive Costs and Life–History Trade-Offs

Our findings identify a significant “cost of mating,” as virgin individuals consistently exhibited superior survival under starvation compared to their mated counterparts. This observation aligns with the Disposable Soma Theory, illustrating a fundamental life–history trade-off where the preferential allocation of limited metabolic resources toward reproductive effort occurs at the expense of somatic maintenance and stress resilience [13,35]. Notably, in specific cohorts—particularly protein-fed males—the physiological impact of mating on hazard rates surpassed that of chronological age.

In the context of the Teneral Reserve Hypothesis, our results suggest that the depletion of endogenous resources acquired during the pre-adult stages is accelerated by reproductive activity. For males, the substantial energetic investment in courtship, intra-sexual competition, and the synthesis of accessory gland fluids [12,36,37] appears to exhaust these finite teneral reserves prematurely. Interestingly, mating in males appears to override the influence of senescence; mated males on a full diet showed no significant survival variance across age groups. This suggests that the energetic drain of mating is sufficiently severe to reach a physiological “survival floor,” neutralizing any age-related advantages in resource sequestration.

Similarly, females incur exhaustive costs through oogenesis and the mechanical stressors of the mating process [37,38,39,40]. Under the framework of the Teneral Reserve Hypothesis, the diversion of lipids and proteins from somatic storage to egg production significantly diminishes the buffer required to withstand starvation. Consequently, the interaction between reproductive investment and the exhaustion of initial reserves serves as a primary driver of physiological decline in tephritids.

### 4.3. Dietary Modulation and the “Protein-Survival” Trade-Off

Dietary composition significantly modulated the capacity of *B. oleae* to endure starvation. While a protein-rich diet is essential for oogenesis and nitrogen exploitation in this species [39], our findings suggest that high-quality nutrient intake is necessary to build the physiological buffer required for starvation resistance. The observation that a restricted (sugar-only) diet enhances starvation resistance, particularly in young virgin females, aligns with the Nutritional Geometry framework. This suggests that a reduction in protein intake, relative to carbohydrate consumption, can trigger a physiological shift toward somatic maintenance, effectively optimizing survival when total nutrient availability is subsequently withdrawn [38].

Sugar-fed (restricted diet) females likely stayed in a “survival mode,” preserving their lipid bodies for a longer duration, whereas protein consumption may trigger a physiological shift toward reproduction at the expense of longevity. This reflects species-specific metabolic strategies where dietary quality directly influences the accumulation and mobilization of energy reserves [41,42].

### 4.4. Sex-Specific Physiological Plasticity

Sex-specific differences were evident, with females exhibiting a more consistent and steep age-related decline in resistance compared to males. This dimorphism likely stems from the massive resource allocation required for egg production. Research suggests that while females may maintain higher baseline levels of lipids and glycogen (“physiological capital”) [33,43], the metabolic overhead of being “pro-ovigenic” (maintaining reproductive readiness) is higher than the maintenance costs in males [40].

As females age, the continuous investment in reproductive preparation likely accelerates the depletion of the fat body, leaving them more vulnerable to starvation. Conversely, the more volatile resistance observed in males suggests a higher vulnerability to rapid reserve depletion during active courtship, necessitating further research into sex-specific metabolic rates.

### 4.5. Boundaries and Future Avenues

Despite the robust initial sample size (*N* = 150 per treatment group), certain subgroups exhibited very large hazard ratios accompanied by wide confidence intervals in the Cox proportional hazards analysis. This numerical instability is a recognized limit in survival models when event density is low within specific strata, particularly in groups with high survival rates where deaths are sparse or clustered at late time points. As visualized in the Forest Plot (Figure 7), while the precision of these estimates varies, the directional effect remains consistent across all biological contexts, with hazard ratios consistently aligning to the right of the null line. While these values clearly indicate the direction and statistical significance of the aging effect, the precise magnitude of the hazard ratios in these specific cases should be interpreted with caution.

While the present study offers a high-resolution analysis of starvation resistance, certain boundaries regarding physiological and temporal resolution should be noted. First, the interpretations concerning resource allocation and somatic reserves are based on phenotypic observations and life–history theory rather than direct biochemical quantification. Although starvation resistance is a widely recognized proxy for metabolic reserves in tephritid ecology, the absence of direct lipid or glycogen measurements means that the causal links remain inferred.

Furthermore, regarding the selection of age intervals, we utilized three discrete classes (15, 30, and 45 days) as representative physiological milestones of the *B. oleae* life cycle. Although our survival data were recorded at high-resolution (4-h intervals), a continuous daily monitoring of survival curves from day 15 through day 45 would have introduced prohibitive computational and analytical complexity, likely obscuring the primary effects of senescence. By grouping data into these critical stages, we maintained the necessary statistical power to perform a robust Cox Proportional Hazards analysis and to derive clear, interpretable hazard ratios that characterize the transition from peak reproduction to advanced senescence.

Finally, while these findings might offer valuable insights into pest management strategies such as the Sterile Insect Technique (SIT), the results should be interpreted with caution, as laboratory-based starvation treatments represent controlled conditions that may only partially reflect the complex, multi-stress scenarios encountered in the field. Future research should prioritize the biochemical quantification of lipid and glycogen mobilization rates to further elucidate the functional link between survival dynamics and the depletion of internal reserves identified in this study.

## 5. Conclusions

This investigation delineates the physiological constraints governing starvation resistance in the olive fruit fly, *B. oleae*, establishing that stress tolerance is a plastic trait shaped by the convergence of senescence, reproductive effort, and nutritional history. Our findings, supported by robust survival-based modeling and hazard analysis, provide a critical basis for understanding how this species manages finite energy budgets under acute environmental stress, leading to the following conclusions:

- Senescence as a driver of physiological decay: Age serves as the fundamental determinant of survival. The observed decline in starvation resistance post-maturation indicates a progressive loss of homeostatic competence and the exhaustion of physiological buffers. The 15-day-old cohort represents the peak of somatic resilience, whereas senescent individuals (45 days old) exhibit a steep reduction in probability of survival, indicating consistent with the progressive depletion of endogenous reserves and potentially reflecting a low evolutionary potential for extending starvation resistance in this specialized tephritid.
- The metabolic cost of reproduction: Mating status acts as a potent physiological stressor that frequently surpasses the impact of chronological age on mortality rates. The rapid depletion of metabolic reserves in mated individuals underscores a critical life–history trade-off. In this “disposable soma” framework, the diversion of energy toward courtship and reproductive behaviors occurs at the direct expense of somatic maintenance and survival during nutrient deprivation.
- Nutritional modulation and resilience: Adult dietary quality dictates the accumulation and mobilization of metabolic reserves, demonstrating how nutritional geometry and the balance of macronutrients shape survival trajectories. While protein-rich diets are necessary for high fecundity, they also modulate the fly’s ability to withstand starvation. High-quality nutrition provides the precursors for a robust metabolic buffer in virgin adults; however, the restriction of sugar exacerbates the energetic debt incurred during mating, leading to accelerated mortality.
- Sex-specific patterns in energy allocation: Distinct survival trajectories exist between the sexes. Females maintain a more regulated decline in resistance throughout senescence, likely due to a larger initial “reserve capital.” In contrast, males exhibit higher vulnerability to rapid reserve depletion, reflecting the volatile metabolic costs associated with male-specific reproductive strategies.

From an ecological and applied perspective, integrating these physiological insights with further research provides a strategic pathway toward more effective and environmentally friendly control methods. By identifying specific windows of physiological vulnerability, we can optimize the timing of interventions and refine population dynamics models. This knowledge is particularly vital for the enhancement of the Sterile Insect Technique (SIT) and other biological control strategies, reducing reliance on chemical insecticides and promoting sustainable integrated pest management (IPM) in olive groves. Future research should prioritize the biochemical quantification of lipid and glycogen mobilization rates to further elucidate the functional link between survival dynamics and the depletion of internal reserves.

## Figures and Tables

**Table 1:** Descriptive statistics of the treatments.

Sex	Diet	Mating Status	Age Groups	Mean ± SE	95% Confidence Interval	
					Lower Bound	Upper Bound
Male	Full	Virgin	15	72.2 ± 3.8	64.654	79.666
			30	50.4 ± 3.0	44.498	56.325
			45	43.6 ± 2.3	39.187	48.025
		Mated	15	33.5 ± 3.7	26.215	40.785
			30	25.2 ± 2.3	20.721	29.701
			45	28.2 ± 2.8	22.601	33.724
	Restricted	Virgin	15	67.8 ± 2.5	62.953	72.607
			30	50.2 ± 5.9	38.640	61.737
			45	63.3 ± 3.6	56.275	70.362
		Mated	15	63.9 ± 3.5	57.091	70.709
			30	41.7 ± 3.9	34.137	49.263
			45	35.7 ± 3.6	28.589	42.793
Female	Full	Virgin	15	72.2 ± 2.1	67.997	76.363
			30	56.0 ± 2.5	51.024	60.887
			45	49.5 ± 1.5	46.607	52.393
		Mated	15	50.8 ± 3.5	44.002	57.638
			30	40.9 ± 3.2	34.769	47.120
			45	30.7 ± 3.0	24.743	36.657
	Restricted	Virgin	15	104.2 ± 3.8	96.787	111.680
			30	68.7 ± 5.1	58.667	78.689
			45	83.1 ± 5.2	72.826	93.336
		Mated	15	79.6 ± 6.9	66.174	93.086
			30	51.8 ± 4.6	42.813	60.742
			45	38.6 ± 3.1	32.638	44.645

**Table 2:** Log-Rank (Mantel–Cox) results in comparing the survival data across the 8 treatments.

Treatments	*N* of Insects	χ^2^ (Log-Rank)	*df*	*p*
Male, full diet, virgin	150	32.608	2	<0.001
Male, full diet, mated	150	4.197	2	0.123
Male, restricted diet, virgin	150	3.805	2	0.149
Male, restricted diet, mated	150	18.471	2	<0.001
Female, full diet, virgin	150	39.808	2	<0.001
Female, full diet, mated	150	15.256	2	<0.001
Female, restricted diet, virgin	150	19.439	2	<0.001
Female, restricted diet, mated	150	23.858	2	<0.001

Note: To address multiple testing, a Bonferroni-adjusted *p*-value of 0.00625 was used as the criterion for statistical significance.

## Data Availability

Data available on request from the corresponding author due to intellectual property restrictions of the Laboratory of Applied Zoology and Parasitology in Agriculture, School of Agriculture, Aristotle University of Thessaloniki.

## Abbreviations

The following abbreviations are used in this manuscript:

*B*	The estimated regression coefficient
$e^{B}$ = *Exp*(*B*)	Hazard Ratio

## Appendix A

**Table A1:** Comparative analysis of survival metrics across treatments by age group. The Change (%*Δ*) column represents the percentage change in the hazard of mortality for the 30-day-old and 45-day-old cohorts relative to the 15-day-old reference group, as well as for the 30-day-old cohort relative to the 45-day-old reference group.

Treatments	Age Group (Days)	Reference Age	*B*	SE	Wald	*p*	HR *Exp*(*B*)	Change (%*Δ*)	95% CI (Lower)	95% CI (Upper)
V_F_♂	30	15	2.00	0.55	13.33	0.000	7.383	+638.3%	2.52	21.59
	45	15	2.56	0.52	24.57	0.000	12.896	+1189.6%	4.69	35.45
	30	45	−0.56	0.42	1.74	0.187	0.573	−42.7%	0.25	1.31
V_F_♀	30	15	3.64	1.08	11.36	0.001	37.915	+3691.5%	4.58	314.05
	45	15	4.75	1.13	17.65	0.000	115.263	+11,426.3%	12.58	1055.86
	30	45	−1.11	0.51	4.83	0.028	0.329	−67.1%	0.12	0.89
V_R_♂	30	15	0.82	0.44	3.58	0.059	2.282	+128.2%	0.97	5.36
	45	15	0.21	0.37	0.33	0.567	1.235	+23.5%	0.60	2.55
	30	45	0.61	0.43	1.99	0.158	1.847	+84.7%	0.79	4.33
V_R_♀	30	15	2.04	0.51	16.24	0.000	7.673	+667.3%	2.85	20.67
	45	15	0.86	0.38	5.00	0.025	2.352	+135.2%	1.11	4.98
	30	45	1.18	0.46	6.55	0.011	3.262	+226.2%	1.32	8.07
M_F_♂	30	15	1.01	0.52	3.78	0.052	2.754	+175.4%	0.99	7.65
	45	15	0.63	0.52	1.44	0.230	1.870	+87.0%	0.67	5.19
	30	45	0.39	0.50	0.60	0.437	1.473	+47.3%	0.55	3.91
M_F_♀	30	15	0.77	0.50	2.42	0.120	2.164	+116.4%	0.82	5.72
	45	15	1.98	0.57	12.11	0.001	7.244	+624.4%	2.37	22.10
	30	45	−1.21	0.53	5.22	0.022	0.299	−70.1%	0.11	0.84
M_R_♂	30	15	1.59	0.55	8.35	0.004	4.898	+389.8%	1.67	14.39
	45	15	2.24	0.58	14.92	0.000	9.391	+839.1%	3.01	29.26
	30	45	−0.65	0.48	1.88	0.171	0.522	−47.8%	0.21	1.32
M_R_♀	30	15	1.56	0.61	6.50	0.011	4.756	+375.6%	1.43	15.77
	45	15	2.78	0.67	17.23	0.000	16.173	+1517.3%	4.34	60.20
	30	45	−1.22	0.51	5.67	0.017	0.294	−70.6%	0.11	0.81

Where: *B* (Coefficient): The estimated regression coefficient, *Exp*(*B*) = $e^{B}$ (Hazard Ratio): The exponentiated coefficient, representing the Hazard Ratio. It indicates the relative likelihood of the event (mortality) occurring in the 30-day and 45-day groups compared to the 15-day reference group, as well as in the 30-day group compared to the 45-day reference group. Abbreviations: V: virgin; M: mated; F: full diet; R: restricted diet, ♂: males, ♀: females.

Note on Model Validity: The overall statistical significance of the Cox regression models was verified using the Omnibus Test of Model Coefficients. In specific cohorts where Hazard Ratios appear exceptionally high with wide confidence intervals (e.g., V_F_♀, HR = 115.263), these values reflect extreme biological divergence in survival probability under stress rather than model failure. Such numerical instability is expected in strata with high survival rates and low event density. To ensure robustness, model stability was further confirmed through the inclusion of time-dependent covariates, which showed no significant violation of the proportional hazard’s assumption for these groups (*p* > 0.05).

**Table A2:** Statistical evaluation of the proportional hazard’s assumption using time-dependent covariates for each experimental group.

Treatments	−2 Log Likelihood	Overall (Score)			Change From Previous Step			Change From Previous Block		
		Chi-Square	*df*	Sig.	Chi-Square	*df*	Sig.	Chi-Square	*df*	Sig.
V_F_♂	149.842	92.545	53	0.001	71.221	53	0.048	71.221	53	0.048
V_F_♀	220.641	112.823	48	0.000	0.000	48	1.000	0.000	48	1.000
V_R_♂	147.178	78.663	73	0.304	73.707	73	0.455	73.707	73	0.455
V_R_♀	220.641	98.081	61	0.002	0.000	61	1.000	0.000	61	1.000
M_F_♂	87.297	39.257	36	0.326	42.660	36	0.206	42.660	36	0.206
M_F_♀	88.314	55.134	33	0.009	49.049	33	0.036	49.049	33	0.036
M_R_♂	91.209	67.283	47	0.028	58.108	47	0.128	58.108	47	0.128
M_R_♀	110.175	60.264	35	0.005	46.649	35	0.090	46.649	35	0.090

Where: V: virgin; M: mated; F: full diet; R: restricted diet, ♂: males, ♀: females.

**Table A3:** Tests of Between—Subjects Effects.

Sex		Type III Sum of Squares	*df*	Mean Square	*F*	Sig.	Partial Eta Squared	Noncent. Parameter	Observed Power ^b^
Male	Corrected Model	32,448.577 ^a^	11	2949.871	21.067	0.000	0.650	231.740	1.000
	Intercept	296,194.899	1	296,194.899	2115.355	0.000	0.944	2115.355	1.000
	V_M	12,722.599	1	12,722.599	90.862	0.000	0.421	90.862	1.000
	F_R	4321.213	1	4321.213	30.861	0.000	0.198	30.861	1.000
	15–30–45	8794.934	2	4397.467	31.406	0.000	0.334	62.811	1.000
	V_M × F_R	1381.121	1	1381.121	9.864	0.002	0.073	9.864	0.876
	V_M × 15–30–45	135.926	2	67.963	0.485	0.617	0.008	0.971	0.128
	F_R × 15–30–45	177.201	2	88.600	0.633	0.533	0.010	1.266	0.154
	V_M × F_R × 15–30–45	3314.522	2	1657.261	11.836	0.000	0.159	23.672	0.994
	Error	17,502.668	1788	140.021					
	Total	398,417.000	1800						
	Corrected Total	49,951.246	1799						
Female	Corrected Model	63,140.974 ^c^	11	5740.089	31.875	0.000	0.734	350.625	1.000
	Intercept	480,560.739	1	480,560.739	2668.577	0.000	0.955	2668.577	1.000
	V_M	18,148.711	1	18,148.711	100.781	0.000	0.442	100.781	1.000
	F_R	14,455.903	1	14,455.903	80.274	0.000	0.387	80.274	1.000
	15–30–45	18,993.974	2	9496.987	52.737	0.000	0.454	105.474	1.000
	V_M × F_R	863.001	1	863.001	4.792	0.030	0.036	4.792	0.584
	V_M × 15–30–45	1318.414	2	659.207	3.661	0.028	0.055	7.321	0.665
	F_R × 15–30–45	1811.574	2	905.787	5.030	0.008	0.073	10.060	0.808
	V_M × F_R × 15–30–45	1037.677	2	518.838	2.881	0.060	0.043	5.762	0.555
	Error	22,870.323	1788	180.081					
	Total	639,114.400	1800						
	Corrected Total	86,011.298	1799						

Where: (1) mating status: V: virgin, M: mated; (2) diet: F: full, R: restricted; (3) age groups: 15-, 30-, and 45-day-old. ^a^ R^2^ = 0.650 (Adjusted R^2^ = 0.619). ^b^ Computed using α = 0.05, ^c^ R^2^ = 0.734 (Adjusted R^2^ = 0.711).

**Table A4:** Treatment comparisons with biologically and statistically significant differences.

					95% Confidence Interval		
Treatment i	Treatment j	Mean Difference (i-j)	Std. Error	*p*	Lower Bound	Upper Bound	Statistically Significant Difference in
V_F_♂_15	V_F_♂_30	21.749 *	4.99	0.002	5.13	38.37	age group
V_F_♂_15	V_F_♂_45	28.554 *	4.25	0.000	14.39	42.72	age group
V_F_♂_15	M_F_♂_15	38.660 *	4.83	0.000	22.57	54.75	mating status
V_F_♂_30	M_F_♂_30	25.200 *	5.58	0.001	6.62	43.78	mating status
V_R_♂_45	V_F_♂_45	19.712 *	4.18	0.000	33.65	5.78	diet
V_R_♂_15	V_R_♂_30	17.591 *	4.99	0.028	0.97	34.21	age group
V_R_♂_45	M_R_♂_45	27.628 *	4.63	0.000	12.19	43.07	mating status
M_R_♂_15	M_F_♂_15	30.400 *	5.29	0.000	12.77	48.03	diet
M_R_♂_15	M_R_♂_30	22.200 *	5.44	0.004	4.09	40.31	age group
M_R_♂_15	M_R_♂_45	28.209 *	5.17	0.000	10.99	45.43	age group
V_F_♀_15	V_F_♀_45	22.680 *	4.82	0.000	6.62	38.74	age group
V_R_♀_15	V_F_♀_15	32.053 *	4.90	0.000	48.37	15.74	mating status
V_F_♀_15	M_F_♀_15	21.360 *	5.48	0.008	3.12	39.60	mating status
V_F_♀_45	M_F_♀_45	18.800 *	5.59	0.046	0.18	37.42	mating status
V_R_♀_15	V_R_♀_30	35.556 *	5.66	0.000	16.71	54.40	age group
V_R_♀_15	V_R_♀_45	21.152 *	4.82	0.001	5.09	37.21	age group
V_R_♀_45	V_F_♀_45	33.581 *	4.74	0.000	17.78	49.38	diet
V_R_♀_15	M_R_♀_15	24.603 *	5.48	0.001	6.36	42.85	mating status
V_R_♀_45	M_R_♀_45	44.440 *	5.12	0.000	27.38	61.50	mating status
M_R_♀_15	M_F_♀_15	28.810 *	6.00	0.000	48.79	8.83	diet
M_R_♀_15	M_R_♀_30	27.852 *	6.17	0.001	7.32	48.38	age group
M_R_♀_15	M_R_♀_45	40.988 *	5.75	0.000	21.86	60.12	age group

Where: (1) mating status: V: virgin, M: mated; (2) diet: F: full, R: restricted; (3) sex: ♂: males, ♀: females; (4) age groups: 15-, 30-, and 45-day-old.
